# Effects of CYP3A5 Polymorphism on Rapid Progression of Chronic Kidney Disease: A Prospective, Multicentre Study

**DOI:** 10.3390/jpm11040252

**Published:** 2021-03-30

**Authors:** Fei Yee Lee, Farida Islahudin, Aina Yazrin Ali Nasiruddin, Abdul Halim Abdul Gafor, Hin-Seng Wong, Sunita Bavanandan, Shamin Mohd Saffian, Adyani Md Redzuan, Nurul Ain Mohd Tahir, Mohd Makmor-Bakry

**Affiliations:** 1 Centre for Quality Management of Medicines, Faculty of Pharmacy, Universiti Kebangsaan Malaysia, Kuala Lumpur 50300, Malaysia; feiyee7890@gmail.com (F.Y.L.); aina@cyberjaya.edu.my (A.Y.A.N.); shamin@ukm.edu.my (S.M.S.); adyani@ukm.edu.my (A.M.R.); nurulainmt@ukm.edu.my (N.A.M.T.); mohdclinpharm@ukm.edu.my (M.M.-B.); 2 Clinical Research Centre, Hospital Selayang, Ministry of Health Malaysia, Batu Caves, Selangor 60800, Malaysia; hinseng@gmail.com; 3 Faculty of Pharmacy, University of Cyberjaya, Cyberjaya 63000, Malaysia; 4 Nephrology Unit, Department of Medicine, Universiti Kebangsaan Malaysia Medical Centre, Kuala Lumpur 56000, Malaysia; halimgafor@gmail.com; 5 Nephrology Department, Hospital Selayang, Ministry of Health Malaysia, Batu Caves, Selangor 60800, Malaysia; 6 Nephrology Department, Hospital Kuala Lumpur, Ministry of Health Malaysia, Kuala Lumpur 50586, Malaysia; sbavanandan@gmail.com

**Keywords:** pharmacogenomics, clinical translation, chronic kidney disease, CYP3A5, polymorphism, progression

## Abstract

Personalised medicine is potentially useful to delay the progression of chronic kidney disease (CKD). The aim of this study was to determine the effects of CYP3A5 polymorphism in rapid CKD progression. This multicentre, observational, prospective cohort study was performed among adult CKD patients (≥18 years) with estimated glomerular filtration rate (eGFR) ≥30 mL/min/1.73 m^2^, who had ≥4 outpatient, non-emergency eGFR values during the three-year study period. The blood samples collected were analysed for CYP3A5*3 polymorphism. Rapid CKD progression was defined as eGFR decline of >5 mL/min/1.73 m^2^/year. Multiple logistic regression was then performed to identify the factors associated with rapid CKD progression. A total of 124 subjects consented to participate. The distribution of the genotypes adhered to the Hardy–Weinberg equilibrium (X^2^ = 0.237, *p* = 0.626). After adjusting for potential confounding factors via multiple logistic regression, the factors associated with rapid CKD progression were CYP3A5*3/*3 polymorphism (adjusted Odds Ratio [aOR] 4.190, 95% confidence interval [CI]: 1.268, 13.852), adjustments to antihypertensives, young age, dyslipidaemia, smoking and use of traditional/complementary medicine. CKD patients should be monitored closely for possible factors associated with rapid CKD progression to optimise clinical outcomes. The CYP3A5*3/*3 genotype could potentially be screened among CKD patients to offer more individualised management among these patients.

## 1. Introduction

Chronic kidney disease (CKD) is a rising public health problem with an alarming increasing trend [1]. During management of CKD patients, optimal control is important to delay disease progression [2]. The control of progression among CKD patients is very often reliant on various pharmacological treatments, such as antihypertensives and antidiabetic drugs, to manage complications associated with kidney failure. However, pharmacotherapy requires close monitoring in order to delay rapid progression of CKD. Rapid progression of CKD is associated with poorer clinical outcomes, including cardiovascular events and death, irrespective of renal function [3]. Recently, optimising management has been focused on personalised treatment, involving identification of interpatient variability, as well as optimisation of treatment effectiveness and safety based on pharmacogenetic data [4].

Pharmacogenetic differences in the cytochrome P (CYP) 450 system have been of interest, as CYP450 enzymes metabolise more than 80% of all prescribed drugs [5]. One of the most common CYP450 enzymes among Asians is the CYP3A5, in which the single nucleotide polymorphisms (SNP) were found in 65.7–71.3% of the Asian population [6]. Genetic polymorphism of CYP3A5 affects the quantity of the functioning enzyme, which then potentially affects the metabolism of various drugs [7]. Interindividual variations of the *CYP3A5* gene expression occur with the presence of the CYP3A5*3 allele, which causes a replacement of adenine (A) by a guanine (G), at position 6986 of the intron 3, creating a cryptic splice site that causes a premature stop codon, leading to the absence of the CYP3A5 protein [7,8]. Thus, individuals with the CYP3A5*3 allele tend to express a lesser amount of CYP3A5 enzyme [7]. Despite the highly polymorphic nature of *CYP3A5* gene with variants from CYP3A5*1 to CYP3A5*9, CYP3A5*3 polymorphism (rs776746, RefSeq NG_007938.2:g.12083A>G) is the most commonly reported CYP3A5 polymorphism in almost all populations [6].

Potentially, the CYP3A5*3 polymorphism could affect both antihypertensive management as well as blood pressure control [8,9,10,11,12]. Approximately 90% of CKD patients are treated with antihypertensive agents, of which pharmacogenetics have been shown to influence outcomes [13]. Interestingly, CYP3A5*3 polymorphism has been found to be associated with variability in blood pressure response to several calcium-channel blockers, such as amlodipine [8], felodipine [9], diltiazem [10] and verapamil [11,12]. In contrast, the influence of CYP3A5*3 pharmacogenetics was found to be lacking towards first-line antihypertensives for CKD, namely angiotensin converting enzyme inhibitor (ACEI) and angiotensin II blocker (ARB) [6]. Apart from the potential influence on drug-metabolising properties, the polymorphism of the *CYP3A5* gene has also been studied previously for its role in blood pressure control [8]. In animals, CYP3A5 enzymes have been shown to convert cortisol to 6β-hydroxycortisol, followed by promotion of post-renal proximal tubular sodium reabsorption, water retention and elevation of blood pressure [8].

Hypertension is a known consequence, as well as a cause, of CKD. Studies have shown that CKD progression is notably accelerated when blood pressure is sustainably high [3]. CYP3A5 activity may be related to the pathogenesis of CKD progression, through reduction of the renin-angiotensin-aldosterone system (RAAS) activity [14]. The CYP3A5 enzyme may reduce RAAS activity by converting corticosterone to 6β-hydroxycorticosterone instead of aldosterone, which subsequently reduces the aldosterone-induced RAAS activity [8]. This is also supported by the findings of a recent study involving CKD patients that showed 20-hydroxyeicosatetraenoic (HETE) acid, a product of CYP enzyme, is a predictor of CKD progression [15].

The possible link of CYP3A5 activity with blood pressure control, drug-metabolising activity of CYP3A5 and CKD progression have highlighted the potential role of CYP3A5 pharmacogenetics in optimising therapeutic management. Therapeutic outcomes could be better if monitoring based on CYP3A5-polymorphism status could be conducted, to adjust for the unexpected effects of medications driven by the SNP. The association between CYP3A5*3 polymorphism and antihypertensive medication, blood pressure control and CKD progression show marked differences in results reported from different ethnicities and geographical locations [8]. Asians have been reported to exhibit faster CKD progression than Caucasians, which was not found to be related to their demographic and clinical characteristics, as well as their laboratory parameters [16]. Therefore, it is increasingly important to investigate genetic factors among this population, to identify potential factors that may contribute to the risk of rapid CKD progression. The impact of CYP3A5 polymorphism in Asians might be more profound than other CYP enzymes, given its higher prevalence than other CYP enzymes [6]. Therefore, the aim of this research was to determine the effects of CYP3A5*3 genetic polymorphisms in rapid CKD progression among an Asian population of CKD patients with routine nephrology care.

## 2. Materials and Methods

### 2.1. Study Design

This multicentre, observational, prospective cohort study was performed among adult CKD patients (aged ≥ 18 years), with an estimated glomerular filtration rate (eGFR) of 30 mL/min/1.73 m^2^ and above [3], in three tertiary hospitals with specialist nephrology clinics in Malaysia. The study was approved by the Medical Research Ethics Committee, Malaysia (KKM.NIHSEC.P19-2320(11))and the Universiti Kebangsaan Malaysia Research Ethic Committee (UKM PPI/111/8/JEP-2020-048). This study was conducted in compliance with ethical principles outlined in the Declaration of Helsinki and the Malaysian Good Clinical Practice Guidelines. The study report follows the Strengthening the Reporting Of Pharmacogenetic Studies (STROPS) guidelines [17].

Potentially eligible patients were identified via pre-screening from patient clinic lists and data from medical records. Patients were then recruited by the investigators during clinic visits from March 2020 until September 2020. Written informed consent was obtained from every subject prior to participation in this study. Patients with at least four outpatient visits and non-emergency eGFR values during the three-year study period were recruited, to ensure sufficient information was available to estimate the risk of CKD progression [18]. Patients who were pregnant, lactating, had incomplete medication regimen or without routine nephrology care were excluded.

### 2.2. Data Collection

After informed consent was obtained from subjects, each participant was assigned a unique subject identification number. Subjects’ names were kept on a password-protected database. Demographic data, clinical information, laboratory data and medication characteristics for each subject from January 2018 to December 2020 were collected from the medical records from the respective institutions. Demographic data that were collected were age, sex and ethnicity.

The clinical information included the primary cause of CKD, co-morbidities, obesity (defined as body mass index (BMI) of 30 kg/m^2^ and above), smoking status and blood pressure level, measured during clinic visits. The laboratory data collected were serum creatinine, albuminuria/proteinuria status and haemoglobin level during the study period.

Medication profiles of the subjects, adherence to medications and the use of traditional or complementary medicine (TCM) were compiled from the electronic medical record and prescription data, as well as from a structured interview with the subjects on their medication-taking behaviour, conducted by two investigators using a standardised questionnaire [2]. The data on the number of adjustments to antihypertensives (changes in dosage, frequency, timing or cessation, or commencement of new antihypertensives) were collected.

### 2.3. Study Definitions

Patients’ renal function were quantified via eGFR calculated using the CKD Epidemiology Collaboration (CKD-EPI) equation. The CKD and albuminuria classification was based on the Kidney Disease: Improving Global Outcomes Workgroup (KDIGO) 2012 guidelines [18]. The definitions for each category are detailed in Table 1.

Patients’ renal function were quantified via eGFR calculated using the CKD-EPI equation, using outpatient, non-emergency serum creatinine values [3]. Emergency serum creatinine values were defined as serum creatinine values obtained during visits to an emergency department. These values were excluded to avoid interference of transiently elevated serum creatinine values, due to acute illness or acute kidney injury (AKI) rather than actual CKD progression. The rapid progression of CKD was defined as a sustained decline in eGFR of more than 5 mL/min/1.73 m^2^/year, in line with the KDIGO CKD guidelines [18] (Table 1). AKI was detected using the KDIGO guideline criteria from the serum creatinine values, as well as information about AKI episodes that occurred in other healthcare institutions and were documented in the subjects’ medical records.

Routine nephrology care was defined as documentation of ambulatory nephrology care by a nephrologist in the medical records for at least 5 years [21].

Adjustments to antihypertensives included changes in dosage, frequency and timing, as well as cessation or commencement of new antihypertensives [20].

Adherence to medications was considered to be poor if there was a discrepancy between the prescribers’ order and actual medication taken [19], based on documentations in medical records and patient recall (Table 1).

Consumption of TCM was defined as the use of treatment and medicines not prescribed from hospitals or health clinics, such as the use of herbs (or botanicals), as well as over-the-counter nutritional and dietary supplements, based on patient recall [20].

### 2.4. Sample Size

Prior data indicate that the proportion of the CYP3A5*3/*3 genetic polymorphism status is 0.437 [6] and the population size of eligible patients was 180. If the Type I error probability and precision are 0.05 and 0.05, the sample size is 124 samples [22].

### 2.5. Detection of CYP3A5*3 Gene Polymorphism

Genomic DNA was extracted from blood samples using a DNeasy^®^ Blood and Tissue extraction kit (Qiagen, Hilden, Germany). The extracted genomic DNA was then analysed for purity and measured for concentration through OPTIZEN NanoQ spectrophotometer (Kaia Bio-Ingenieria, Daejeon, Korea), in which the 260/280 absorption ratio between 1.70 to 1.99 was considered to be DNA with sufficient purity without contamination during the extraction process [23]. The extracted genomic DNA was stored at −80 °C until use.

The intron 3 of the CYP3A5 gene encompassing the rs776746 (RefSeq NG_007938.2:g.12083A>G) polymorphism was amplified by the primers 5′-CAGCAAGAGTCTCACACAGG-3′ (Forward) and 5′-TACCACCCAGCTTAACGAAT-3′ (Reverse) (IDT DNA, Singapore) and TopTaq Mastermix Kit (Qiagen, Hilden, Germany) using the Arktik^TM^ Thermal Cycler (Thermo Fisher Scientific, Finland). The polymerase chain reaction (PCR) products were examined by gel electrophoresis to ensure the quality of PCR products through Invitrogen™ 2% E-Gel™ Agarose Gels with SYBR Safe™ (Thermo Fisher Scientific, Kiryat Shmona, Israel) for 26 min, in which the PCR products were segregated by size and captured using the E-Gel^®^ Safe Imager^TM^ Realtime Transilluminator (Life Technologies, Kiryat Shmona, Israel) [24]. The PCR product was then purified by a commercialised PCR purification kit (Applied Biosystems, UK) as a precondition for DNA Sanger sequencing. Purified DNA fragments were analysed using the BigDye^®^ Terminator version 3.1 cycle sequencing kit, which were run on a 96-capillary 3730xl DNA Analyzer at First BASE Laboratories Sdn. Bhd., Malaysia (developed by Applied Biosystem, USA, and produced by Thermo Fisher Scientific). The laboratory personnel were blinded, such that they were unable to distinguish samples with or without rapid CKD progression. The DNA sequences of the SNP results were transcribed using Sequence Scanner version 2.0 software (Applied Biosystems) and checked with the reference sequences in the Basic Local Alignment Search Tool (BLAST) program to confirm the presence of the polymorphism [25].

### 2.6. Statistical Analysis

All statistics were performed using IBM Statistical Package for Social Science for Windows version 23 (IBM Corp, Armonk, NY, USA). The results are presented as frequencies and percentages for categorical data, mean ± standard deviation (SD) for normally distributed numerical data, or as median (range) for non-normally distributed numerical data, based on the inspection of histograms. Adherence of the genotype groups to the Hardy-Weinberg equilibrium (HWE) assumption was examined. Expected percentages for each genotype group were calculated based on the Hardy-Weinberg equation using the allele frequencies (p^2^ + 2pq + q^2^ = 1). Chi-square test was then used to compare the allele and genotype distribution found with the predicted distribution. The observed genotype distribution was considered to be consistent with the assumptions of HWE if the *p*-value >0.05 [24]. An independent T-test was used to compare the normally distributed numerical data between two groups, while the Mann-Whitney U test was used to compare the non-normally distributed numerical data between two groups. One-way ANOVA test was used to analyse normally distributed numerical data for comparison of more than two groups. Pearson’s Chi-square test for independence was used to study the association between categorical data and categorical data, while Fisher’s exact test was used if assumptions of Pearson’s Chi-square test for independence were not met.

To investigate the relationship between CYP3A5 polymorphism and rapid CKD progression among the study population, linear regression was first performed using outpatient, non-emergency serum creatinine values over 3 years, to quantify the eGFR slope of each subject to identify subjects with rapid CKD progression [3]. Multiple logistic regression was then applied to identify the factors associated with rapid CKD progression, as the assumptions to perform a linear regression were not met. A simple logistic regression was performed with each independent variable, to determine factors at a level of significance of *p* ≤ 0.05 [26]. A multiple stepwise logistic regression was then performed with all factors with *p* < 0.25, in which variables with *p* ≤ 0.05 were considered as factors associated with rapid progression of CKD, followed by an examination of multicollinearity and interaction between these factors, by a Variance Inflation Factor (VIF) of 5 and above defined as presence of multicollinearity [27]. The Hosmer-Lemeshow goodness-of-fit test, classification tables and area under the receiving operator characteristic (ROC) curve were used to investigate any misrepresentation of data [26].

## 3. Results

### 3.1. Demographic and Clinical Characteristics

From 180 potentially eligible patients, a total of 124 subjects were included, with an average age of 52.2 ± 15.7 years, equal distribution of sex (*n* = 62, 50.0%) and predominantly Malay ethnicity (*n* = 71, 57.3%) (Table 2). Twenty-nine of the 124 subjects (23.4%) were found to have rapid CKD progression. The median eGFR decline per year for rapid CKD progressors was 6.0 mL/min/1.73 m^2^/year (range: −5.06 to −32.65 mL/min/1.73 m^2^/year). For non-rapid CKD progressors, the median eGFR decline per year was 0.86 mL/min/1.73 m^2^/year (range: −4.98 to 8.86 mL/min/1.73 m^2^/year).

Subjects with rapid CKD progression had a median of 3 (range: 0–19) adjustments to antihypertensives throughout the study period, which was significantly higher than subjects without rapid CKD progression, with a median of 1 (range: 0–15) adjustment (*p* = 0.001). Cessation of RAAS blockade occurred in 6 (6.3%) patients without rapid CKD progression and 3 (10.3%) patients with rapid CKD progression. TCM use was reported among 6 (20.7%) subjects with rapid CKD progression and 10 (10.5%) subjects without rapid CKD progression.

### 3.2. Allele and Genotype Analysis

Each participant’s genotype was analysed to detect the presence of CYP3A5*3 (rs776746, RefSeq NG_007938.2:g.12083A>G) polymorphism, and was compared with the rate of eGFR decline. The proportion of CYP3A5*3 allele was found to be 23.8% (*n* = 59), while the proportion of the wildtype allele was 76.2% (*n* = 189). Meanwhile, the distribution of CYP3A5*3 allele for each ethnic group was 19.7% (*n* = 28), 30.7% (*n* = 27) and 22.2% (*n* = 4) for Malay, Chinese and Indians, respectively. The distribution of the genotypes fulfilled the assumptions and predicted distribution from the Hardy-Weinberg equation (X^2^ = 0.237, *p* = 0.626) [24].

The baseline eGFR did not differ significantly with variants of CYP3A5 allele (*p* = 0.731) nor genotypes (*p* = 0.438) (Table 3). By the end of the study period, the average eGFR among subjects with the CYP3A5*3/*3 genotype of 45.7 ± 20.9 mL/min/1.73 m^2^ was significantly lower than subjects with CYP3A5*1/*1 genotype (58.2 ± 32.6 mL/min/1.73 m^2^) or CYP3A5*1/*3 genotype (63.3 ± 34.7 mL/min/1.73 m^2^) (*p* = 0.030); while there was no statistically significant difference between subjects in terms of the allelic frequency (*p* = 0.862). Baseline albuminuria status did not differ significantly with variants of CYP3A5 allele (*p* = 1.000) nor genotype (*p* = 0.487). By the end of the study period, the distribution of albuminuria status was significantly different by allele (*p* = 0.007), as well as by genotype (p = 0.029) (Table 3). From the perspective of genotype, subjects with CYP3A5*1/*1 genotype had a decline in the number of A3 albuminuria status by the end of the study period, from 28 (38.4%) to 22 (30.1%). Subjects with CYP3A5*1/*3 genotype had more A3 albuminuria status by the end of the study period, from 15 (34.9%) to 21 (48.8%), while subjects with CYP3A5*3/*3 genotype had the highest proportion of patients with A3 albuminuria category at baseline and at the end of the study period (*n* = 4, 50%). The number of patients with CYP3A5*1 allele in A1 category declined from 74 (39.2%) to 62 (32.8%) by the end of the study period. For subjects with CYP3A5*3 allele, A3 category patients increased from 23 (39.0%) to 29 (49.2%) by the end of the study period.

Twenty-nine (23.4%) subjects had rapid CKD progression, with 4 (13.8%) having the CYP3A5*3/*3 genotype. From the remaining 95 patients without rapid CKD progression, 4 (4.2%) patients had CYP3A5*3/*3 genotype, while 4 (13.8%) patients with rapid CKD progression had CYP3A5*3/*3 genotype.

### 3.3. Factors Associated with Rapid CKD Progression

Table 4 shows the factors associated with rapid CKD progression. The simple logistic regression showed that adjustments to antihypertensives, CYP3A5*3/*3 polymorphism, previous episode of AKI, smoking and use of TCM were factors associated with rapid CKD progression, with a significance level of *p* < 0.05. After adjusting for potential confounding factors with *p* < 0.25 using multiple logistic regression, the factors associated with rapid CKD progression were adjustments to antihypertensives (adjusted Odds Ratio [aOR] 1.172, 95% confidence interval [CI]: 1.055, 1.301), CYP3A5*3/*3 polymorphism (aOR 4.190, 95% CI: 1.268, 13.852), young age (aOR 0.963, 95% CI: 0.937, 0.989), dyslipidaemia (aOR 2.317, 95% CI: 1.030, 5.211), smoking (aOR 7.126, 95% CI: 2.144, 23.685) and use of TCM (aOR 2.684, 95% CI: 1.045, 6.891) (Table 5).

## 4. Discussion

Rapid progression of CKD occurred in approximately a fifth of our study population with mild-to-moderate CKD within a span of three years. To the best of our ability and knowledge, this is the first study describing the link between CYP3A5 polymorphism and rapid CKD progression. As the HWE assumptions were not violated, systematic error was likely absent in the genotyping assays. After adjusting for potential confounding factors, CYP3A5*3/*3 genotype, adjustments to antihypertensives, young age, dyslipidaemia, smoking and use of TCM were found to be factors associated with rapid CKD progression.

Our study supports previous reports [15,28] that have demonstrated that CYP3A5 polymorphism may be associated with factors of CKD progression. CYP3A5 is not only a drug metabolising enzyme present in the liver, it is also present in the kidneys and might have important physiological functions [7]. The CYP3A5*3 allele is associated with less expression of the CYP3A5 enzyme in the kidneys of healthy human adults compared to the wildtype, CYP3A5*1 [28]. The diminished CYP3A5 activity from the CYP3A5*3/*3 polymorphism reduces the protection against aldosterone-induced active sodium transport in the kidneys, as less intrarenal conversion of the corticosterone into 6β-hydroxycorticosterone occurs [8]. Less inhibition of RAAS, which is related to glomerular hyperfiltration, exacerbates damage to the kidneys. This is supported by our finding that a greater proportion of subjects with CYP3A5*3/*3 genotype had category A3 albuminuria by the end of the study period, than those with CYP3A5*1/*1 or CYP3A5*3/*3 genotype, in line with findings from a previous study in which proteinuria was found to be an indicator of structural kidney damage [3]. Furthermore, maximal doses of ACEI or ARB did not improve renal function among patients with aldosterone excess [29], while CKD patients with aldosterone excess were reported to have accelerated progression of CKD [29].

Another possible mechanism for the effect of CYP3A5*3/*3 genotype on rapid CKD progression is the elevation of 20-HETE production. The arachidonic acid-derived metabolites of CYP3A5 enzyme are 19-HETE and 6β-hydroxycortisol [30]. The CYP3A5*3 polymorphism results in a reduced expression of CYP3A5 enzymes, which in turn reduces the formation of 19-HETE [30]. This may lead to an increased availability of the arachidonic acid precursor for greater production of 20-HETE. The increase of 20-HETE has been shown to increase renal vasoconstriction and peripheral vascular resistance, as 20-HETE is a potent vasoconstrictor that mediates angiotensin II-related renal effects in the proximal tubule and thick ascending limb of the loop of Henle [30]. In addition, a higher level of 20-HETE was recently identified as an independent predictor of CKD progression [15]. This demonstrates the need for more studies to be conducted to elucidate the mechanism of association between CYP3A5*3 polymorphism and rapid CKD progression. Genotyping may be beneficial to identify CKD patients with CYP3A5*3/*3 genotype for closer monitoring, given the association found between CYP3A5*3/*3 genotype and accelerated CKD progression.

There was a significant association between antihypertensive adjustments and rapid CKD progression found in the current work. During early progression, first-line RAAS inhibitors may have been stopped once patients presented with a rapidly deteriorating eGFR, which could account for part of the medication adjustments [31]. On the other hand, genetic predisposition may also account for frequent medication adjustments. Pharmacokinetic properties of CYP3A5 substrate drugs are known to differ according to CYP3A5 polymorphism [8]. In particular, calcium channel blocker antihypertensives, such as amlodipine [8], felodipine [9], diltiazem [10] and verapamil [11,12] have been reported to be affected by CYP3A5 polymorphism, with blood pressure responses varying according to their CYP3A5 polymorphism status [8,11,12]. However, the complexity of genetic effects are evident as metabolism of CYP3A5 substrates among individuals who are CYP3A5 non-expressors (those expressing CYP3A5*3/*3 genotype) have been reported to be carried out by the CYP3A4 enzyme, which is more prone to inductions and inhibitions by concurrent drugs [5]. These genetic effects may have led to the need for frequent medication adjustments for optimum outcome, as observed in the current work, supporting the need for closer monitoring of antihypertensive management.

Younger age and dyslipidaemia were also found to be associated with rapid CKD progression, in line with findings from previous studies on rapid CKD progression [32,33]. It is believed that the CKD aetiology is different in older patients, with more aggressive disease found among younger patients, while in adults some decline of eGFR is believed to occur as part of aging, rather than from deteriorating CKD [34]. On the other hand, in CKD patients, lipoproteins are oxidised, especially the small dense high-density lipoprotein cholesterol (LDL) particles, intermediate-density lipoproteins and chylomicron remnants [35]. Accumulation of these oxidised LDL, intermediate-density lipoproteins and chylomicron remnants accelerates systemic inflammation, through stimulating release of proinflammatory cytokines and chemokines from monocytes and macrophages [35]. The subsequent systemic inflammation and oxidative stress is believed to cause eGFR decline and CKD progression [35].

Smoking was also found to be associated with a higher risk of CKD progression, similar to previous work [36]. The potential mechanisms of smoking-associated CKD progression include smoking-induced hypoxic injury [36], myointimal hyperplasia of intrarenal arterioles [37] and adverse effects on intrarenal hemodynamics, through nicotine-induced release of angiotensin II [36]. This leads to increased activation of RAAS, as well as increased glomerular hypertension, which could potentially accelerate the progression of CKD. The study finding suggests that smoking cessation is an important component to preserve the kidney function of CKD patients who are currently smoking.

The association between TCM and CKD progression in Asian countries has been inconsistent to date [1,38]. A few studies have reported no association between TCM and CKD progression [38,39]. This may be due to the relatively short follow-up period that might not capture renal damage in the long term [38]. On the other hand, the lack of association has also been attributed to TCM that was prescribed by board-certified physicians and produced by pharmaceutical companies, which had certified manufacturing practices [39]. In the current work, we found a significant association between rapid CKD progression and TCM consumption, of which the TCM was used without the supervision of a registered practitioner or pharmacist. It was noted that many reported the use of TCM with unknown ingredients and quality. Most worrying is that some TCM, most often involving the use of herbal remedies popular among Asians, have been shown to contain nephrotoxic ingredients [40]. However, very often CKD patients report the use of TCM due to the lack of conventional medications that cure CKD, as well as the desire to see immediate improvement in their disease condition [40]. As there is no cure for CKD in conventional medicine, some patients might be inclined to use TCM, owing to their cultural beliefs and social influences [40]. Therefore, TCM use for CKD should be monitored closely for mitigation efforts in preventing rapid CKD progression.

There were a few limitations to our findings. Firstly, the findings of the study have limited applicability to advanced CKD patients with Stage 4 and above, in which the majority of these patients were shown to exhibit non-linear CKD progression [32]. In addition, the report of TCM use might be subject to recall bias at the time of interview. The potential effects of such bias were reduced by incorporating the report of TCM use from medical records. The lack of identification of TCM also provides fewer specific details of which moieties were nephrotoxic to the patients. Furthermore, the effects of other potential genes were not studied. As CYP3A4 and CYP3A5 enzymes have some overlapping in substrate specificity, the complete loss of metabolic activity with the CYP3A5*3 allele might pronounce the impact of genetic variation in CYP3A4 expression among these patients [5]. Therefore, further work involving genetic variants of CYP3A4 polymorphism could possibly improve the current findings. The study might be limited by the absence of direct measurement in the expression level or activities of CYP3A5. Future studies could be designed to investigate the activity of CYP3A5 with renal function through usage of endogenous markers, such as 4β-hydroxycholesterol. More studies could be conducted to investigate other outcomes, such as initiation of renal replacement therapy, heart failure and mortality, as well as CKD progression over a longer period of time.

## 5. Conclusions

In conclusion, CKD must be monitored closely to reduce the risk of rapid progression. This could potentially mean monitoring of patient genetics, as the CYP3A5*3/*3 genotype was found to be associated with accelerated CKD progression after adjusting for possible confounding factors. CKD patients with such characteristics, as well as those requiring antihypertensive adjustments, young age, dyslipidaemia, smokers and TCM users, may also benefit from intensified monitoring and care to reduce the propensity of developing adverse clinical outcomes. Most importantly, our findings suggest a potentially important role of CYP3A5 polymorphism in the pathogenesis of accelerated CKD progression. A personalised management approach could therefore be potentially useful for CKD patients based on genotyping data.

## Figures and Tables

**Table 1:** Terminologies and their definitions. pertaining to the study.

Terminology		Definition
Classification of CKD [18]	Stage 1	Normal or elevated GFR, with GFR of 90 mL/min/1.73 m^2^ and above
	Stage 2	Mildly decreased GFR of 60–89 mL/min/1.73 m^2^
	Stage 3a	Mild to moderately decreased GFR of 45–59 mL/min/1.73 m^2^
	Stage 3b	Moderately to severely decreased GFR of 30–44 mL/min/1.73 m^2^
	Stage 4	Severely decreased GFR of 15–29 mL/min/1.73 m^2^
	Stage 5	Low eGFR of less than 15 mL/min/1.73 m^2^
Albuminuria categorisation [18]	A1	Protein-to-creatinine ratio (PCR) of less than 15 mg/mmol and below or negative to trace from urine protein reagent strip
	A2	PCR of 15–50 mg/mmol or trace to + from urine protein reagent strip
	A3	PCR of more than 50 mg/mmol, or greater than + from urine protein reagent strip
Progression of CKD	Rapid CKD progression	Sustained decline in eGFR of more than 5 mL/min/1.73 m^2^/year [18], based on the rate of annual eGFR change using linear regression model to identify the eGFR slope using the eGFR collected during the study period [3]
Types of non-adherence [19]	Initiation phase	Medication is not taken by patient at all
	Implementation phase	A dose is missed, omitted or an extra dose taken
	Persistence phase	The medication is ceased without the instruction of prescriber
Others	TCM consumption	The use of therapies not included in the treatment and medicines prescribed by hospitals or health clinics, such as the use of herbs (or botanicals), as well as over-the-counter nutritional and dietary supplements, based on patient recall [20]

**Table 2:** Demographic and clinical characteristics of subjects.

Characteristics	Non-Rapid CKD Progression (*n* = 95)	Rapid CKD Progression (*n* = 29)	Total (*n* = 124)
Age, mean (SD)	53.2 (15.4)	49.0 (16.2)	52.2 (15.7)
Ethnicity, *n* (%)			
Malay ethnicity, *n* (%)	55 (57.9)	16 (55.2)	71 (57.3)
Others, *n* (%)	40 (42.1)	13 (44.8)	53 (42.7)
Male sex, *n* (%)	46 (48.4)	16 (55.2)	62 (50.0)
CYP3A5 polymorphism, *n* (%)			
*1/*1	58 (61.1)	15 (51.7)	73 (58.9)
*1/*3	33 (34.7)	10 (34.5)	43 (34.7)
*3/*3	4 (4.2)	4 (13.8)	8 (6.5)
Stage of CKD, *n* (%)			
1	28 (29.5)	7 (24.1)	35 (28.2)
2	15 (15.8)	7 (24.1)	22 (17.7)
3a	17 (17.9)	6 (20.7)	23 (18.5)
3b	35 (36.8)	9 (31.0)	44 (35.5)
Baseline albuminuria status, *n* (%)			
A1	41 (43.2)	8 (27.6)	49 (39.5)
A2	17 (17.9)	7 (24.1)	24 (19.4)
A3	35 (36.8)	13 (44.8)	48 (38.7)
Missing	2 (2.1)	1 (3.4)	3 (2.4)
Baseline systolic blood pressure, mmHg, mean (SD)	133.0 (16.7)	135.2 (19.3)	133.6 (17.3)
CVD, *n* (%)	13 (13.7)	6 (20.7)	19 (15.3)
CCF, *n* (%)	6 (6.3)	1 (3.4)	7 (5.6)
Diabetes, *n* (%)	32 (33.7)	11 (37.9)	43 (34.7)
Dyslipidaemia, *n* (%)	60 (63.2)	22 (75.9)	82 (66.1)
Episode of AKI, *n* (%)	8 (8.4)	6 (20.7)	14 (11.3)
Gout, *n* (%)	23 (24.2)	6 (20.7)	29 (23.4)
Obesity (BMI > 30 kg/m^2^), *n* (%)	12 (12.6)	6 (20.7)	18 (14.5)
Anaemia, *n* (%)	36 (37.9)	13 (44.8)	49 (39.5)
Smoking status, *n* (%)			
Non-smoker	88 (92.6)	23 (79.3)	111 (89.5)
Ex-smoker	4 (4.2)	2 (6.9)	6 (4.8)
Currently smoking	3 (3.2)	4 (13.8)	7 (5.6)
Uncontrolled hypertension, *n* (%)	71 (77.2)	23 (79.3)	94 (77.7)
Adjustments to antihypertensives, median (range)	1 (0–15)	3 (0–19)	2 (0–19)
Poor medication adherence, *n* (%)	37 (38.9)	13 (44.8)	50 (40.3)
Use of calcium channel blockers, *n* (%)	55 (57.9)	20 (69.0)	75 (60.5)
Cessation of RAAS blockade, *n* (%)	6 (6.3)	3 (10.3)	9 (7.3)
Use of TCM, *n* (%)	10 (10.5)	6 (20.7)	16 (12.9)

AKI, acute kidney injury; BMI, body mass index; CCF, congestive cardiac failure; CKD, chronic kidney disease, CVD, cardiovascular disease; eGFR, estimated glomerular filtration rate; SD, standard deviation; TCM, traditional/complementary medicine.

**Table 3:** Renal function stratified by allele and genotype distribution.

Variables	Allele (*n* = 248)			Genotype (*n* = 124)			
	CYP3A5*1 (Wildtype)	CYP3A5*3 (Variant)	*p*-Value	Homozygous Wild Type (*1/*1)	Heterozygous (*1/*3)	Homozygous (*3/*3)	*p*-Value
Baseline eGFR, mL/min/1.73 m^2^, mean (SD)	66.5 (33.0)	64.9 (32.0)	0.731 ^a^	66.1 (32.9)	68.0 (33.5)	56.5 (25.8)	0.438 ^c^
eGFR at 3 years, mL/min/1.73 m^2^**,** mean (SD)	59.4 (33.1)	58.5 (32.5)	0.862 ^a^	58.2 (32.6)	63.3 (34.7)	45.7 (20.9)	0.030 ^d^
Baseline albuminuria status, *n* (%)							0.487 ^e^
A1	74 (39.2)	24 (40.7)	1.000 ^b^	29 (39.7)	16 (37.2)	4 (50.0)	
A2	37 (19.6)	12 (20.3)		13 (17.8)	12 (27.9)	-	
A3	72 (38.1)	23 (39.0)		28 (38.4)	15 (34.9)	4 (50.0)	
Missing	6 (3.2)	-		3 (4.1)	-	-	
Albuminuria category at 3 years, *n* (%)							0.029 ^e^
A1	62 (32.8)	22 (37.3)	0.007 ^b^	24 (32.9)	14 (32.6)	4 (50.0)	
A2	61 (32.3)	7 (11.9)		27 (37.0)	7 (16.3)	-	
A3	65 (34.4)	29 (49.2)		22 (30.1)	21 (48.8)	4 (50.0)	
Missing	1 (0.5)	1 (1.7)		-	1 (2.3)	-	

^a^ Independent T-Test; ^b^ Chi-square Test; ^c^ ANOVA test; ^d^ Welch’s ANOVA test as the variances were unequal; ^e^ Fisher’s exact test.

**Table 4:** Factors associated with rapid CKD progression (simple logistic regression).

Variables (Reference)	b	Odds Ratio (95% CI)	*p*-Value
Adjustments to antihypertensives	0.176	1.192 (1.086, 1.309)	<0.001
Age, years	−0.017	0.983 (0.964, 1.002)	0.074
Anaemia of Hb < 13 g/dL (No anaemia)	0.286	1.332 (0.735, 2.414)	0.345
Baseline eGFR, mL/min/1.73 m^2^	0.003	1.003 (0.994, 1.012)	0.583
Baseline albuminuria status (A1)			
A2	0.747	2.110 (0.928, 4.797)	0.075
A3	0.644	1.904 (0.946, 3.832)	0.071
Baseline systolic blood pressure, mmHg	0.009	1.009 (0.992, 1.026)	0.303
CYP3A5*3 (CYP3A5*1) allele	0.492	1.635 (0.850, 3.148)	0.141
Cardiovascular disease (No cardiovascular disease)	0.498	1.645 (0.771, 3.512)	0.198
Congestive cardiac failure (No Congestive cardiac failure)	−0.635	0.530 (0.115, 2.439)	0.415
CYP3A5 polymorphism (CYP3A5*1/*1) *1/*3 *3/*3			
	0.158	1.172 (0.617, 2.225)	0.628
	1.352	3.867 (1.341, 11.150)	0.012
Diabetes (No diabetes)	0.185	1.203 (0.654, 2.214)	0.552
Dyslipidaemia (No dyslipidaemia)	0.606	1.833 (0.938, 3.582)	0.076
Ethnicity (Malay) Others			
	0.111	1.117 (0.618, 2.020)	0.714
Gout (Absence of gout)	−0.203	0.817 (0.399, 1.672)	0.817
Male sex (Female sex)	0.271	1.311 (0.726, 2.366)	0.369
Obesity (No obesity)	0.590	1.804 (0.839, 3.882)	0.131
Occurrence of AKI (No AKI)	1.043	2.837 (1.255, 6.415)	0.012
Poor medication adherence (Good adherence)	0.242	1.274 (0.703, 2.307)	0.425
Smoking status (Non-smoker) Former smoker Current smoker			
	0.649	1.913 (0.552, 6.633)	0.307
	1.630	5.101 (1.686, 15.435)	0.004
Use of TCM (Did not use TCM)	0.796	2.217 (1.010, 4.868)	0.047
Use of calcium channel blockers (Did not use calcium channel blockers)	0.480	1.616 (0.864, 3.024)	0.133
Cessation of RAAS blockade (None)	0.537	1.712 (0.613, 4.782)	0.305
Uncontrolled hypertension (None)	0.126	1.134 (0.550, 2.335)	0.733

**Table 5:** Factors associated with rapid CKD progression (multiple logistic regression).

Variables (Reference)	b	Adjusted Odds Ratio (95% CI)	*p*-Value ^a^
Age, years	−0.038	0.963 (0.937, 0.989)	0.013
Adjustments to antihypertensives	0.158	1.172 (1.055, 1.301)	0.003
CYP3A5 polymorphism (CYP3A5*1/*1) *1/*3 *3/*3			
	0.052	1.053 (0.509, 2.181)	0.889
	1.433	4.190 (1.268, 13.852)	0.019
Dyslipidaemia (No dyslipidaemia)	0.840	2.317 (1.030, 5.211)	0.042
Smoking status (Non-smoker) Former smoker Current smoker			
	1.016	2.763 (0.717, 10.650)	0.140
	1.964	7.126 (2.144, 23.685)	0.001
Use of TCM (Did not use TCM)	0.987	2.684 (1.045, 6.891)	0.040

^a^ Stepwise multiple logistic regression model was applied. Multicollinearity and interaction terms were checked and not found. Hosmer-Lemeshow test (*p* = 0.352), classification table (overall correctly classified percentage = 79.0%) and area under the ROC curve (77.4%) were applied to check the model fit.

## Data Availability

Authors do not have permission to share the data. The data underlying the results presented in the study are available upon request from corresponding author (faridaislahudin@ukm.edu.my) for researchers who meet the criteria for access to confidential data.

## References

[1] Saminathan T.A., Hooi L.S., Mohd Yusoff M.F., Ong L.M., Bavanandan S., Rodzlan Hasani W.S., Tan E.Z.Z., Wong I., Rifin H.M., Robert T.G. (2020). Prevalence of chronic kidney disease and its associated factors in Malaysia; Findings from a nationwide population-based cross-sectional study. BMC Nephrol..

[2] Islahudin F., Lee F.Y., Tengku Abd Kadir T.N.I., Abdullah M.Z., Makmor-Bakry M. (2021). Continuous medication monitoring: A clinical model to predict adherence to medications among chronic kidney disease patients. Res. Soc. Adm. Pharm..

[3] Go A.S., Yang J., Tan T.C., Cabrera C.S., Stefansson B.V., Greasley P.J., Ordonez J.D., Kaiser Permanente Northern California CKD Outcomes Study (2018). Contemporary rates and predictors of fast progression of chronic kidney disease in adults with and without diabetes mellitus. BMC Nephrol..

[4] Adams S.M., Crisamore K.R., Empey P.E. (2018). Clinical pharmacogenomics. Clin. J. Am. Soc. Nephrol..

[5] Zanger U.M., Schwab M. (2013). Cytochrome P450 enzymes in drug metabolism: Regulation of gene expression, enzyme activities, and impact of genetic variation. Pharmacol. Ther..

[6] Dorji P.W., Tshering G., Na-Bangchang K. (2019). CYP2C9, CYP2C19, CYP2D6 and CYP3A5 polymorphisms in South-East and East Asian populations: A systematic review. J. Clin. Pharm. Ther..

[7] Kuehl P., Zhang J., Lin Y., Lamba J., Assem M., Schuetz J., Watkins P.B., Daly A., Wrighton S.A., Hall S.D. (2001). Sequence diversity in CYP3A promoters and characterization of the genetic basis of polymorphic CYP3A5 expression. Nat. Genet..

[8] Zhang Y.P., Zuo X.C., Huang Z.J., Cai J.J., Wen J., Duan D.D., Yuan H. (2014). CYP3A5 polymorphism, amlodipine and hypertension. J. Hum. Hypertens..

[9] Xiang Q., Li C., Zhao X., Cui Y.M. (2017). The influence of CYP3A5*3 and BCRPC421A genetic polymorphisms on the pharmacokinetics of felodipine in healthy Chinese volunteers. J. Clin. Pharm. Ther..

[10] Zhou L.-Y., Zuo X.-C., Chen K., Wang J.-L., Chen Q.-J., Zhou Y.-N., Yuan H., Ma Y., Zhu L.-J., Peng Y.-X. (2016). Significant impacts of CYP3A4*1G and CYP3A5*3 genetic polymorphisms on the pharmacokinetics of diltiazem and its main metabolites in Chinese adult kidney transplant patients. J. Clin. Pharm. Ther..

[11] Jin Y., Wang Y.H., Miao J., Li L., Kovacs R.J., Marunde R., Hamman M.A., Philips S., Hilligoss J., Hall S.D. (2007). Cytochrome P450 3A5 genotype is associated with verapamil response in healthy subjects. Clin. Pharmacol. Ther..

[12] Langaee T.Y., Gong Y., Yarandi H.N., Katz D.A., Cooper-DeHoff R.M., Pepine C.J., Johnson J.A. (2007). Association of CYP3A5 polymorphisms with hypertension and antihypertensive response to verapamil. Clin. Pharmacol. Ther..

[13] Schmidt I.M., Hübner S., Nadal J., Titze S., Schmid M., Bärthlein B., Schlieper G., Dienemann T., Schultheiss U.T., Meiselbach H. (2019). Patterns of medication use and the burden of polypharmacy in patients with chronic kidney disease: The German Chronic Kidney Disease study. Clin. Kidney J..

[14] Rüster C., Wolf G. (2006). Renin-Angiotensin-aldosterone system and progression of renal disease. J. Am. Soc. Nephrol..

[15] Afshinnia F., Zeng L., Byun J., Wernisch S., Deo R., Chen J., Hamm L., Miller E.R., Rhee E.P., Fischer M.J. (2018). Elevated lipoxygenase and cytochrome P450 products predict progression of chronic kidney disease. Nephrol. Dial. Transplant..

[16] Barbour S.J., Er L., Djurdjev O., Karim M., Levin A. (2010). Differences in progression of CKD and mortality amongst Caucasian, Oriental Asian and South Asian CKD patients. Nephrol. Dial. Transpl..

[17] Chaplin M., Kirkham J.J., Dwan K., Sloan D.J., Davies G., Jorgensen A.L. (2020). STrengthening the reporting of pharmacogenetic studies: Development of the STROPS guideline. PLoS Med..

[18] KDIGO W.G.C. (2012). KDIGO 2012 clinical practice guideline for the evaluation and management of chronic kidney disease. Kidney Int. Suppl..

[19] Vrijens B., De Geest S., Hughes D.A., Przemyslaw K., Demonceau J., Ruppar T., Dobbels F., Fargher E., Morrison V., Lewek P. (2012). A new taxonomy for describing and defining adherence to medications. Br. J. Clin. Pharmacol..

[20] Lee F.Y., Islahudin F., Makmor-Bakry M., Wong H.-S., Bavanandan S. (2021). Factors associated with the frequency of antihypertensive drug adjustments in chronic kidney disease patients: A multicentre, 2-year retrospective study. Int. J. Clin. Pharm..

[21] Jones C., Roderick P., Harris S., Rogerson M. (2006). Decline in kidney function before and after nephrology referral and the effect on survival in moderate to advanced chronic kidney disease. Nephrol. Dial. Transpl..

[22] Lemeshow S., Hosmer D.W., Klar J., Lwanga S.K. (1990). World Health Organization. Adequacy of Sample size in Health Studies/Stanley Lemeshow.

[23] Lucena-Aguilar G., Sánchez-López A.M., Barberán-Aceituno C., Carrillo-Ávila J.A., López-Guerrero J.A., Aguilar-Quesada R. (2016). DNA source selection for downstream applications based on DNA quality indicators analysis. Biopreserv. Biobank..

[24] Tahir N.A.M., Saffian S.M., Islahudin F.H., Gafor A.H.A., Othman H., Manan H.A., Makmor-Bakry M. (2020). Effects of CST3 Gene G73A Polymorphism on Cystatin C in a Prospective Multiethnic Cohort Study. Nephron.

[25] Ariffin N.M., Islahudin F., Kumolosasi E., Makmor-Bakry M. (2019). Effects of MAO-A and CYP450 on primaquine metabolism in healthy volunteers. Parasitol. Res..

[26] Hosmer D.W.J., Lemeshow S., Hosmer D.W.J., Lemeshow S. (2000). Assessing the fit of the model. Applied Logistic Regression.

[27] Shrestha N. (2020). Detecting multicollinearity in regression analysis. Am. J. Appl. Math. Stat..

[28] Givens R.C., Lin Y.S., Dowling A.L., Thummel K.E., Lamba J.K., Schuetz E.G., Stewart P.W., Watkins P.B. (2003). CYP3A5 genotype predicts renal CYP3A activity and blood pressure in healthy adults. J. Appl. Physiol..

[29] Schjoedt K.J., Andersen S., Rossing P., Tarnow L., Parving H.H. (2004). Aldosterone escape during blockade of the renin-angiotensin-aldosterone system in diabetic nephropathy is associated with enhanced decline in glomerular filtration rate. Diabetologia.

[30] Knights K.M., Rowland A., Miners J.O. (2013). Renal drug metabolism in humans: The potential for drug-endobiotic interactions involving cytochrome P450 (CYP) and UDP-glucuronosyltransferase (UGT). Br. J. Clin. Pharmacol..

[31] Higuchi S., Kohsaka S., Shiraishi Y., Katsuki T., Nagatomo Y., Mizuno A., Sujino Y., Kohno T., Goda A., Yoshikawa T. (2019). Association of renin-angiotensin system inhibitors with long-term outcomes in patients with systolic heart failure and moderate-to-severe kidney function impairment. Eur. J. Int. Med..

[32] Caravaca-Fontán F., Azevedo L., Luna E., Caravaca F. (2018). Patterns of progression of chronic kidney disease at later stages. Clin. Kidney J..

[33] Ali I., Chinnadurai R., Ibrahim S.T., Green D., Kalra P.A. (2020). Predictive factors of rapid linear renal progression and mortality in patients with chronic kidney disease. BMC Nephrol..

[34] O’Hare A.M., Choi A.I., Bertenthal D., Bacchetti P., Garg A.X., Kaufman J.S., Walter L.C., Mehta K.M., Steinman M.A., Allon M. (2007). Age affects outcomes in chronic kidney disease. J. Am. Soc. Nephrol..

[35] Tsuruya K., Yoshida H., Nagata M., Kitazono T., Iseki K., Iseki C., Fujimoto S., Konta T., Moriyama T., Yamagata K. (2015). Impact of the triglycerides to high-density lipoprotein cholesterol ratio on the incidence and progression of CKD: A longitudinal study in a large Japanese population. Am. J. Kidney Dis..

[36] Lee S., Kang S., Joo Y.S., Lee C., Nam K.H., Yun H.-R., Park J.T., Chang T.I., Yoo T.-H., Kim S.W. (2020). Smoking, smoking cessation, and progression of chronic kidney disease: Results from KNOW-CKD study. Nicotine Tob. Res..

[37] Lhotta K., Rumpelt H.J., König P., Mayer G., Kronenberg F. (2002). Cigarette smoking and vascular pathology in renal biopsies. Kidney Int..

[38] Tangkiatkumjai M., Boardman H., Praditpornsilpa K., Walker D.M. (2015). Association of herbal and dietary supplements with progression and complications of chronic kidney disease: A prospective cohort study. Nephrol. (Carlton).

[39] Lin M.-Y., Chiu Y.-W., Chang J.-S., Lin H.-L., Lee C.T.-C., Chiu G.-F., Kuo M.-C., Wu M.-T., Chen H.-C., Hwang S.-J. (2015). Association of prescribed Chinese herbal medicine use with risk of end-stage renal disease in patients with chronic kidney disease. Kidney Int..

[40] Saeed S., Islahudin F., Makmor-Bakry M., Redzuan A.M. (2018). The practice of complementary and alternative medicine among chronic kidney disease patients. J. Adv. Pharm. Edu. Res..

